# Incidence of New Fractures in Patients Treated with Kyphoplasty/Vertebroplasty or Conservative Methods

**DOI:** 10.3390/medicina61112012

**Published:** 2025-11-11

**Authors:** Alper Tabanli, Hakan Yilmaz, Hüseyin Berk Benek, Mehmet Akif Ercan, Gulsen Ozgenc, Cafer Ak, Onur Bologur, Emrah Akcay, Alaettin Yurt

**Affiliations:** 1 Department of Neurosurgery, Faculty of Medicine, Izmir Tinaztepe University, Izmir 35400, Turkey; 2 Department of Neurosurgery, Izmir State Hospital, Izmir 35590, Turkey

**Keywords:** osteoporotic vertebral compression fracture, kyphoplasty, vertebroplasty, conservative treatment, fracture recurrence risk factors

## Abstract

*Background and Objectives:* Osteoporotic vertebral compression fractures (OVCFs) are a common cause of morbidity in the elderly, often resulting in pain, reduced mobility, and diminished quality of life. Treatment options include conservative management, vertebroplasty (VP), and kyphoplasty (KP). This study aimed to compare the clinical outcomes and complication rates of patients treated with kyphoplasty/vertebroplasty versus conservative methods and identify risk factors associated with the development of new fractures. *Materials and Methods:* This retrospective cohort study included patients diagnosed with OVCFs who were treated either surgically (KP/VP) or conservatively between January 2020 and January 2025. Inclusion criteria encompassed vertebral height loss on CT, STIR hyperintensity on MRI, and a T-score below −2.5. Patients were followed for at least one year. Clinical evaluations included pain scores (VAS), functional status (ODI), and quality of life assessments. Complications and new fracture rates were recorded. Logistic regression analysis was performed to identify risk factors influencing fracture recurrence. *Results:* A total of 132 patients were analyzed: 65 in the KP/VP group and 67 in the conservative treatment group. The KP/VP group achieved better postoperative pain results (3.2 ± 1.0 vs. 4.0 ± 1.2) with a significant difference of −0.8 (95% CI: −1.2 to −0.4, *p* = 0.032) and better mobility results (ODI: 4.5 ± 0.8 vs. 3.9 ± 0.9) with a significant difference of 0.6 (95% CI: 0.3–0.9, *p* = 0.047) and improved quality of life scores (6.7 ± 1.1 vs. 5.9 ± 1.3) with a significant difference of 0.8 (95% CI: 0.4–1.2, *p* = 0.041). The incidence of new fractures was similar between groups (15.4% vs. 17.9%, *p* = 0.678). Overall complication rates were 7.7% in the KP/VP group versus 11.9% in the conservative group (*p* = 0.435). The results from logistic regression analysis showed that age (adjusted OR: 2.48, 95% CI: 1.20–5.13), low bone mineral density (adjusted OR: 0.31, 95% CI: 0.15–0.63), and cement leakage (adjusted OR: 3.10, 95% CI: 1.21–7.99) were identified as risk factors for new fractures. The study found that outdoor activity (adjusted OR: 0.38, 95% CI: 0.20–0.73) and anti-osteoporosis treatment (adjusted OR: 0.17, 95% CI: 0.04–0.79) acted as protective factors against new fractures. The KP/VP group required half the time to recover from their injuries because they used their braces for 3.0 ± 0.5 months instead of 6.0 ± 1.0 months (*p* < 0.001). *Conclusions*: Kyphoplasty and vertebroplasty were more effective than conservative treatment in improving pain, mobility, and quality of life in patients with OVCFs. Although the incidence of new fractures did not differ significantly between groups, surgical treatment demonstrated lower complication rates and significantly faster recovery, as evidenced by reduced brace use duration. These findings support the use of KP/VP as a viable option for managing OVCFs in appropriately selected patients.

## 1. Introduction

Osteoporotic vertebral compression fractures (OVCFs) are a prevalent issue in the elderly population and may lead to significant morbidity or mortality. Treatment options include conservative management, vertebroplasty (VP), and kyphoplasty (KP). The efficacy and safety of these modalities have been extensively examined in the literature [1,2]. OVCFs are frequently observed in older individuals and substantially impair quality of life. These fractures result in severe back pain, restricted mobility, and deformity, thereby adversely affecting daily activities. If left untreated, they may cause serious health complications and increase the risk of mortality [3,4]. The limited scope of studies on the effectiveness of OVCF treatment necessitates further investigation. Therefore, evaluating the clinical outcomes and complication rates of VP/KP is of critical importance [5,6].

Our primary hypothesis is that patients undergoing KP/VP will demonstrate superior outcomes in pain management and functional recovery compared to those receiving conservative treatment. Supporting hypotheses include potential differences in the rates of recurrent fractures and complications between KP and VP [7,8]. Although the current literature includes studies comparing the efficacy and safety of VP/KP, most such studies have focused on short-term outcomes and failed to adequately explore long-term clinical results and complication rates [9,10]. Moreover, studies investigating risk factors for new fracture development remain limited, indicating a need for further research [11,12].

Research studies have established vital information about how KP and VP perform against each other. KP shows better results for vertebral height restoration and kyphotic angle correction (*p* < 0.05), yet VP and KP produce equivalent pain relief results with VAS score decreases of 4.6–4.7 points [3]. The two procedures deliver equivalent pain relief results to patients because VAS scores decrease by 4.6–4.7 points [3]. The two procedures, PVP and PKP, achieve substantial pain relief by decreasing VAS scores from 8.0 to 2.7–2.8 after surgery, and these results persist in the long term [5]. The procedure of PVP provides better results through shorter surgical duration, minimal blood loss, and decreased radiation exposure [5]. The safety data show PKP produces fewer cement leaks than PVP (OR = 3.03; 95% CI: 1.58–5.82; *p* < 0.001), yet both methods result in similar new vertebral fracture occurrences at 30–33% [3,7]. The SpineJack implant demonstrates excellent radiological results and minimal complications while showing better performance than other vertebral augmentation devices [6]. The research indicates KP and VP deliver safe and effective results, but doctors should choose between them depending on the specific fracture characteristics [7].

The purpose of this study is to compare the clinical outcomes and complication rates of patients treated with KP/VP and conservative methods, thereby assessing the effectiveness of these treatment modalities. Additionally, logistic regression analysis was employed to identify risk factors associated with new fracture development. The objective is to generate data that will inform the selection of the most appropriate approach for OVCF treatment.

## 2. Materials and Methods

In this study, the efficacy and safety of kyphoplasty (KP), vertebroplasty (VP), and conservative treatment methods in managing osteoporotic vertebral compression fractures (OVCFs) were evaluated. The patient selection and follow-up protocols were adapted based on methods described in previous prospective cohort studies [5]. Imaging modalities included computed tomography (CT) and magnetic resonance imaging (MRI). Participants were selected from patients treated at University of Health Sciences, Bozyaka Training and Research Hospital who met specific inclusion criteria. These criteria comprised vertebral height loss on CT, hyperintensity on STIR-sequence MRI, and a T-score below −2.5. Patients with non-pathological thoracic or lumbar fractures and follow-up periods exceeding one year were included. Both conservatively treated and surgically treated patients were enrolled. The exclusion criteria comprised pathological fractures, infections, neurological deficits or paraplegia, and irregular follow-up attendance. Basic demographic data such as age, sex, height, and weight were recorded for each patient. Additional baseline characteristics, including fracture location (thoracic, thoracolumbar junction, lumbar), number of fractured levels, duration from injury to treatment, and comorbidities, were documented to ensure group comparability.

Sample size calculation was performed based on the primary outcome of VAS pain score reduction. With an expected difference of 1.0 points between groups (SD = 1.2), alpha = 0.05, and power = 80%, a minimum of 60 patients per group was required. Accounting for a 10% dropout, we recruited 65 and 67 patients in the KP/VP and conservative groups, respectively.

These criteria ensured the formation of a homogenous sample aligned with the objectives of the study and minimized the influence of potential confounding variables. Each patient underwent detailed pre- and post-treatment evaluations, and demographic data were systematically documented. The study was designed as a retrospective cohort study comparing the effectiveness of KP/VP and conservative treatment methods. Although randomization and blinding techniques were not applied, comparisons between treatment modalities and outcomes were conducted using objective criteria and standardized protocols. A rigorous methodology was adopted in patient selection and data analysis to improve the reliability and validity of the study. This approach aimed to yield more definitive conclusions regarding the efficacy and safety of the examined treatment methods.

The decision between kyphoplasty and vertebroplasty was based on established morphological criteria [7]. The medical team performed kyphoplasty on patients who had lost more than 30% of their vertebral height and showed potential for height recovery, but used vertebroplasty for patients with fractures that caused less than 30% height loss. The medical team performed the procedures through either CT guidance (*n* = 40, 61.5%) or fluoroscopic guidance (*n* = 25, 38.5%) based on surgeon preference and equipment availability. The medical team recorded all technical details from the procedures, including cement amount, injection force, and the decision between single-sided or dual-sided cement injection.

The patients in the KP/VP group underwent surgical intervention and were monitored at 1, 3, 6, and 12 months in the postoperative period. The pain management protocol provided both groups with standard analgesic treatment through acetaminophen 500 mg taken three times daily and NSAIDs as needed. During follow-up visits, the sudden onset of back pain prompted radiographic and MRI evaluations to detect potential new vertebral fractures. MRI was not performed routinely, but only when patients presented with new or worsening back pain symptoms. This symptom-driven imaging approach may have missed asymptomatic compression fractures, which is acknowledged as a study limitation. These methods facilitated the close monitoring of the postoperative status and complications of each patient. The study defined new fractures as those that occurred either next to the treated vertebra (within two levels) or at a distance of more than two levels from the treated vertebra through radiographic height loss > 20% and MRI signal changes.

The patients in the conservative treatment group did not undergo surgery but received various non-invasive interventions to manage pain and improve mobility, including analgesics, physiotherapy regimens, and brace use. The medical team followed protocol to stop brace use when X-ray images showed bone healing and when patients no longer experienced pain (VAS < 3). The effectiveness of conservative methods was compared to that of surgical interventions.

For each patient, demographic data (age, sex, height, weight) and preoperative bone mineral density (BMD) values were recorded. Clinical outcomes were assessed based on postoperative pain levels, mobility improvements, and quality of life scores. Pain and quality of life were measured using the Visual Analog Scale (VAS; VAS-pain and VAS-QoL, respectively), and mobility was evaluated using the Oswestry Disability Index (ODI). Complication rates were also meticulously followed and recorded. These complications included hospital readmission, infection, and new fracture development. These comprehensive assessments provided detailed and reliable data on the efficacy and safety of the examined treatment methods.

The SPSS software version 26.0 (IBM Corp., Armonk, NY, USA) was used for data analysis. Differences between demographic and clinical variables were assessed using t-tests and chi-squared tests. Mixed-effects models for repeated measures were used to analyze VAS and ODI scores over time, with treatment group, time (categorical), and group × time interaction as fixed effects, and patient as a random effect. Missing data were handled using maximum likelihood estimation under the missing at random assumption. Estimated marginal means and 95% confidence intervals were calculated for all time points.

For the multivariable logistic regression analysis, an exploratory approach was adopted to balance Type I and Type II errors. Only risk factors with *p* < 0.20 in univariable analysis were included in the multivariable model. Backward stepwise selection was employed with entry and retention significance levels of 0.20. Risk factor selection was also guided by biological plausibility and prior knowledge. Multicollinearity was assessed using variance inflation factors (VIF), with VIF > 5 indicating problematic collinearity. The adjusted odds ratios and 95% confidence intervals were calculated for each risk factor in the final model. Logistic regression analysis was employed to identify risk factors influencing new fracture development. The Kaplan–Meier survival analysis method determined the median duration until new fracture occurrence. A significance level of *p* < 0.05 was adopted for all analyses.

## 3. Results

The demographic and clinical characteristics of the patients in the KP/VP and conservative treatment groups were compared. The mean age in the KP/VP group was 70.2 ± 8.5, while it was 71.3 ± 9.0 in the conservative treatment group. In the KP/VP and conservative treatment groups, the proportion of female patients was 56.9% and 55.2%, and body mass index (BMI) values were 25.4 ± 3.6 and 24.9 ± 3.8, respectively. Bone mineral density (BMD) values were −2.8 ± 0.5 in the KP/VP group and −2.7 ± 0.6 in the conservative treatment group. The proportion of patients with a history of falls was 23.1% in the KP/VP group and 26.9% in the conservative treatment group. Fracture location was thoracolumbar junction (T11-L2) in 73.8% of the KP/VP and 71.6% of the conservative group. Mean duration from injury to treatment was 5.2 ± 2.1 days in the KP/VP group and 5.8 ± 2.4 days in the conservative group (*p* = 0.124). The number of fractured levels was 1.3 ± 0.5 in KP/VP and 1.4 ± 0.6 in the conservative group (*p* = 0.298) (Table 1).

According to the analysis of clinical outcomes and complication rates, the hospital readmission rate was 7.7% [95% CI: 2.5–17.0%] in the KP/VP group and 10.4% [95% CI: 4.3–20.3%] in the conservative treatment group, while infection rates were 3.1% [95% CI: 0.4–10.7%] and 1.5% [95% CI: 0.0–8.0%], respectively. The mean postoperative VAS-pain scores were 3.2 ± 1.0 [95% CI: 2.9–3.5] in the KP/VP group and 4.0 ± 1.2 [95% CI: 3.7–4.3] in the conservative treatment group (mean difference: −0.8, 95% CI: −1.2 to −0.4, *p* = 0.032). The mean ODI scores were 4.5 ± 0.8 [95% CI: 4.3–4.7] and 3.9 ± 0.9 [95% CI: 3.7–4.1] in these groups, respectively (mean difference: 0.6, 95% CI: 0.3–0.9, *p* = 0.047). The mean VAS-QoL scores were 6.7 ± 1.1 [95% CI: 6.4–7.0] in the KP/VP group and 5.9 ± 1.3 [95% CI: 5.6–6.2] in the conservative treatment group (mean difference: 0.8, 95% CI: 0.4–1.2, *p* = 0.041). In the KP/VP and conservative treatment groups, the incidence of new fractures was 15.4% [95% CI: 7.6–26.5%] and 17.9% [95% CI: 9.6–29.2%], respectively (*p* = 0.678). The median time to new fracture was 4.5 months (IQR: 3–7) in the KP/VP group and 5.0 months (IQR: 3–8) in the conservative group (*p* = 0.543). Overall complication rates were 7.7% [95% CI: 2.5–17.0%] and 11.9% [95% CI: 5.3–22.2%], respectively (Table 2).

In the multivariable logistic regression analysis, only risk factors with *p* < 0.20 in the univariable analysis were included. Backward stepwise selection was used with entry and retention significance levels of 0.20. Multicollinearity was assessed using variance inflation factors (VIF < 5 for all variables). Age was found to be a significant risk factor for new fractures, with an adjusted odds ratio (OR) of 2.48 (95% CI: 1.20–5.13, *p* = 0.031). Low BMD also significantly increased fracture risk, with an adjusted OR of 0.31 (95% CI: 0.15–0.63, *p* < 0.001). Outdoor activity (ODA) was a protective factor, with an adjusted OR of 0.38 (95% CI: 0.20–0.73, *p* = 0.001). Cement leakage was associated with increased fracture risk, with an adjusted OR of 3.10 (95% CI: 1.21–7.99, *p* = 0.019). Anti-osteoporosis treatment reduced fracture risk, with an adjusted OR of 0.17 (95% CI: 0.04–0.79, *p* = 0.024) (Table 3).

**Figure 1:**
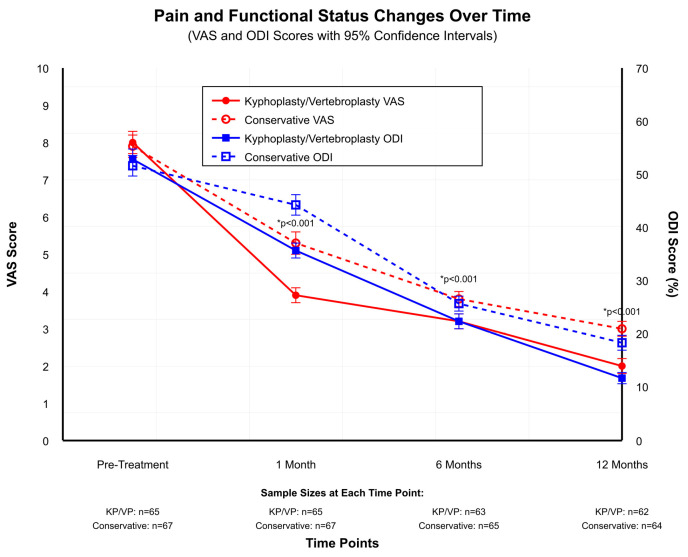
Pain and Functional Status Changes.

Mixed-effects models for repeated measures were used to analyze VAS and ODI scores over time, with group, time, and group × time interaction as predictors. Missing data were handled using maximum likelihood estimation, assuming missing at random. Statistically significant improvements were observed over time in both groups in terms of pain (VAS-pain) and functional status (ODI) scores. In the KP/VP group, the mean VAS-pain score decreased from 8.0 ± 0.9 [95% CI: 7.8–8.2] preoperatively to 2.0 ± 1.4 [95% CI: 1.6–2.4] at month 12 (*p* < 0.001). In the same group, the mean ODI score declined from 58.0 ± 6.2 [95% CI: 56.5–59.5] to 20.0 ± 5.8 [95% CI: 18.6–21.4], indicating a statistically significant improvement (*p* < 0.001). In the conservative treatment group, the mean VAS-pain score decreased from 7.9 ± 0.9 [95% CI: 7.7–8.1] to 3.0 ± 1.4 [95% CI: 2.6–3.4], and the mean ODI score declined from 57.0 ± 6.5 [95% CI: 55.4–58.6] to 27.0 ± 6.2 [95% CI: 25.5–28.5], with both measures showing statistically significant improvement. The analysis showed that the combination of group and time factors produced significant results for both VAS (*p* = 0.018) and ODI (*p* = 0.023), which indicated that the KP/VP group demonstrated quicker recovery. These results suggested that both KP/VP and conservative treatment were effective in pain control and functional recovery. However, the KP/VP group demonstrated superior outcomes in terms of functional improvement compared to the conservative treatment group (Figure 1).

Kyphoplasty was performed in 45 patients (69.2%) and vertebroplasty in 20 patients (30.8%) within the surgical group. Selection was based on fracture morphology: kyphoplasty for fractures with >30% height loss and vertebroplasty for <30% height loss. The CT-guided technique was used in 40 patients (61.5%) and the fluoroscopy-guided technique in 25 patients (38.5%). No significant difference in pain reduction (VAS) or functional improvement (ODI) was observed between CT-guided versus fluoroscopy-guided techniques (*p* = 0.412) or between thoracic and lumbar locations (*p* = 0.523).

**Figure 2:**
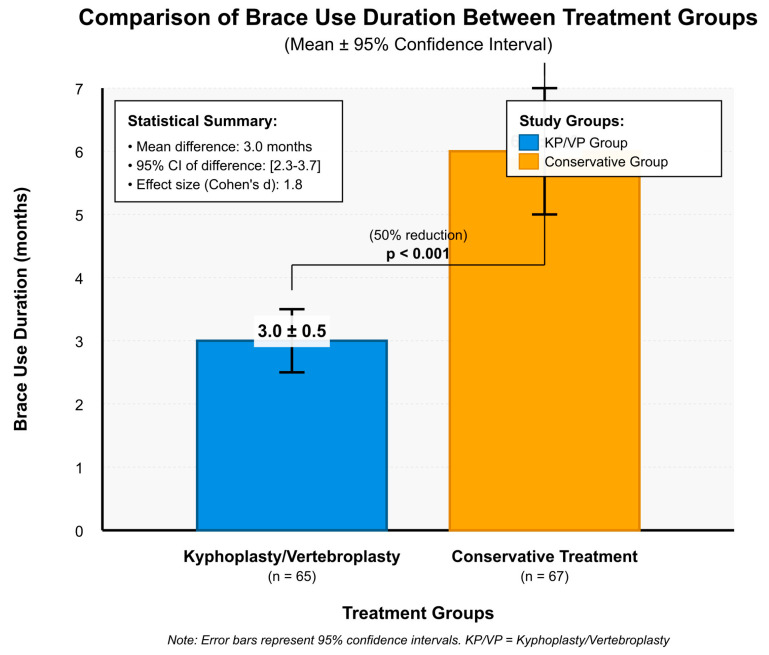
Brace Use Duration.

Brace usage duration was also evaluated. The mean duration in the KP/VP group was 3.0 ± 0.5 months [95% CI: 2.5–3.5], in comparison to 6.0 ± 1.0 months [95% CI: 5.0–7.0] in the conservative treatment group (mean difference: −3.0 months, 95% CI: −3.7 to −2.3, *p* < 0.001), suggesting that KP/VP shortened the need for brace support by 50%. The medical staff followed protocol to stop brace use when patients showed X-ray evidence of bone healing and their pain symptoms disappeared (Figure 2).

## 4. Discussion

The aim of this study was to compare the clinical outcomes and complication rates of patients treated with kyphoplasty/vertebroplasty (KP/VP) and those receiving conservative treatment, thereby evaluating the effectiveness of these treatment modalities. The logistic regression analysis method was also employed to identify risk factors associated with the development of new fractures. In the comparisons of the demographic and clinical characteristics of the KP/VP and conservative treatment groups, no statistically significant differences were observed in terms of age, sex, BMI, BMD, or history of falls. According to the results regarding clinical outcomes and complication rates, the KP/VP group demonstrated a hospital readmission rate of 7.7%, an infection rate of 3.1%, a mean postoperative pain score of 3.2 ± 1.0, a mean mobility improvement score of 4.5 ± 0.8, and a mean quality of life score of 6.7 ± 1.1. In contrast, the conservative treatment group exhibited a hospital readmission rate of 10.4%, an infection rate of 1.5%, a mean pain score of 4.0 ± 1.2, a mean mobility improvement score of 3.9 ± 0.9, and a mean quality of life score of 5.9 ± 1.3. In the logistic regression analysis, risk factors significantly associated with new fracture development included age, low BMD, outdoor activity, cement leakage, and anti-osteoporosis treatment. Graphical evaluations of pain and functional status scores revealed more rapid and pronounced improvements in the KP/VP group. Additionally, brace usage durations were shorter in the KP/VP group. These results indicated that KP/VP was more effective than conservative treatment in managing pain and improving functional outcomes, while also demonstrating lower complication rates.

The data collected in this study were largely consistent with results reported in similar studies conducted in recent years. In the study conducted by Wang et al. [13], the short-term effects of percutaneous curved KP (PCKP) and bilateral percutaneous KP (PKP) were found to be comparable. Similarly, in our study, no statistically significant difference was observed between the KP/VP and conservative treatment groups in terms of the incidence of new fractures. This result indicated that both treatment modalities exhibited similar short-term efficacy [13]. In the study performed by Qi et al. [14], the modified percutaneous KP (MPKP) group demonstrated superior performance compared to the PKP group in terms of vertebral body height restoration and Cobb angle improvement, and the former had lower ODI scores at the final follow-up than the latter. In our study, the KP/VP group also showed better postoperative pain and mobility improvement outcomes compared to the conservative treatment group [14]. Alvi et al. [15] compared the costs and postoperative outcomes of KP and VP. In our study, the KP/VP group exhibited higher quality of life scores than the conservative treatment group. This result supported the notion that KP offers better long-term clinical outcomes [15]. Deng et al. [16] found percutaneous KP (PKP) to be more effective than percutaneous VP (PVP) in the treatment of osteoporotic vertebral asymmetric compression fractures. In our study, the KP/VP group also demonstrated better mobility improvement and quality of life scores [16]. Ge et al. [17] examined the efficacy of percutaneous KP in vertebral compression fractures with varying BMD levels. In our study, low BMD was also identified to raise the risk of new fractures. This result was consistent with the literature, indicating that low BMD is a significant risk factor for the development of new fractures [17]. The data obtained in our study were in agreement with similar studies in the literature and supported the efficacy and safety of KP/VP and conservative treatment modalities. These results suggested that different treatment options should be evaluated based on the demographic characteristics and clinical conditions of patients.

In the study carried out by Wang et al. [13], the short-term clinical outcomes of PCKP and PKP were found to be comparable. Similarly, in our study, the hospital readmission rates in the KP/VP group (7.7%) and the conservative treatment group (10.4%) did not show a statistically significant difference (*p* = 0.548). Infection rates were also consistent with the results reported by Wang et al. [13]. In the study conducted by Qi et al. [14], the MPKP group demonstrated better performance than the PKP group in terms of vertebral body height restoration and Cobb angle improvement, and it had lower ODI values at the final follow-up. In our study, the KP/VP group showed significantly better postoperative pain scores (3.2 ± 1.0) and mobility improvement (4.5 ± 0.8) compared to the conservative treatment group (pain score: 4.0 ± 1.2, mobility improvement: 3.9 ± 0.9) [14]. Deng et al. [16] reported PKP to have better long-term clinical efficacy and lower complication rates compared to PVP. In our study, the overall complication rate in the KP/VP group (7.7%) was lower than that in the conservative treatment group (11.9%) (*p* = 0.435). These results were compatible with the results of Deng et al. [16]. According to Gamal et al. [18], both VP and KP were effective in reducing pain and improving quality of life in OVCFs, with similar postoperative outcomes and complication rates. In our study, the mean quality of life score in the KP/VP group (6.7 ± 1.1) was higher than that in the conservative treatment group (5.9 ± 1.3), and both treatment modalities demonstrated comparable postoperative outcomes [18]. In the study conducted by Yin et al. [19], weight, fracture type, and bone cement distribution were identified as risk factors affecting clinical efficacy in patients treated with KP. In our study, the KP/VP group showed better outcomes in terms of postoperative pain and mobility improvement, while the conservative treatment group exhibited higher complication rates. These results were consistent with the results of Yin et al. [19].

In the study conducted by Mao et al. [20], risk factors associated with secondary fractures included history of additional fractures, age, BMD, bone cement leakage, intravertebral fracture clefts, and Cobb angle. In our study, factors such as age (OR = 2.48, *p* = 0.031), BMD (OR = 0.31, *p* < 0.001), and cement leakage (OR = 3.10, *p* = 0.019) were also found to play a significant role in the development of new fractures. The lack of a significant difference in new fracture rates between KP/VP and conservative groups (15.4% vs. 17.9%, *p* = 0.678) despite superior functional outcomes warrants a biomechanical explanation. The cemented area becomes rigid, which creates a “stiffness mismatch” that produces stress concentration in the surrounding segments [20]. The biomechanical changes from vertebral augmentation procedures might reduce the positive effects of pain relief and functional improvement. In the study carried out by Daher et al. [21], VP and KP were reported to have comparable efficacy in reducing pain and disability. In our study, outdoor activity (OR = 0.38, *p* = 0.001) and anti-osteoporosis treatment (OR = 0.17, *p* = 0.024) were found to reduce the risk of new fractures. The study by Liang et al. showed that preoperative measurements of Adjacent Segment Alignment (ASA) and Thoracolumbar Alignment (TLA) at high levels predict the occurrence of adjacent segment fractures [22]. The thoracolumbar junction between T10 and L2 shows elevated fracture risk because it bears additional loads, and post-vertebroplasty kyphosis creates increased stress on neighboring vertebrae, which results in fracture formation [22]. This result was in agreement with the risk factors identified in our study. In the study conducted by Ye et al. [23], patients with low Hounsfield Unit (HU) values were reported to be at higher risk for new osteoporotic fractures. In our study, BMD was also identified as a significant risk factor for the development of new fractures. The Hounsfield units from CT scans of the vertebrae enable direct trabecular bone density assessment, which does not depend on DXA-measured BMD values. The risk of new fractures after PKP becomes significantly higher when HU values reach below 45–50, which indicates that HU values should be used for preoperative risk assessment [23]. KP was found to be associated with a lower mortality risk in patients with osteoporotic vertebral compression fractures [24]. This result supported the efficacy of KP/VP treatment as demonstrated in our study.

In both groups in our study, significant reductions in pain and improvements in functional status were observed at 1-, 6-, and 12-month follow-ups compared to pre-treatment levels. Nevertheless, the improvements were more pronounced in the KP/VP group. Specifically, in the first month, the mean VAS-pain score in the KP/VP group decreased from approximately 8 to approximately 4. In the conservative treatment group, on the other hand, it decreased from approximately 8 to approximately 5. At the 12-month follow-up, the mean VAS-pain score dropped to approximately 2 in the KP/VP group, whereas it remained at approximately 3 in the conservative treatment group. Mean ODI scores declined from approximately 60 to approximately 20 in the KP/VP group and from approximately 60 to approximately 25 in the conservative treatment group. These results indicated that KP/VP was more effective in both pain management and functional recovery in comparison to conservative treatment.

These results were also supported by similar studies in the literature. For example, Patel et al. [25] reported that balloon KP and VP were effective in reducing pain and improving function in the treatment of vertebral compression fractures. In the study conducted by Lu et al. [26], both PKP and VP were found to be effective in patients with Kümmell’s disease, whereas PKP offered advantages in terms of operative time and blood loss. Zhao et al. [27] reported that PVP significantly improved VAS and ODI scores in patients with chronic OVCFs. Fang et al. [28] found that PKP provided substantial pain relief in patients with thoracolumbar OVCFs and distal lumbosacral pain. Izumida et al. [29] reported that balloon KP effectively reduced severe pain associated with vertebral compression fractures.

The graphical analyses in this study revealed that the patients who underwent KP/VP used braces for shorter periods (3 ± 0.5 months) compared to those who received conservative treatment (6 ± 1 months). The 50% reduction in brace use duration followed protocol guidelines, which used radiographic healing and symptom resolution as criteria instead of patient preference. The shorter recovery period enables patients to start moving again and perform their daily activities sooner. In a large-scale cohort study conducted by Tsai et al. [30], the clinical outcomes of patients treated with VP or KP were evaluated, and it was found that patients who underwent KP achieved better short-term functional outcomes and required a shorter duration of brace use. Liu et al. [31] compared the long-term clinical outcomes of balloon KP and VP and reported that patients treated with KP used a brace for a shorter period and experienced a faster functional recovery than those receiving conservative treatment. Similarly, in the randomized controlled trial conducted by Dohm et al. [32], it was stated that patients treated with KP used a brace for a shorter duration and recovered more quickly than those treated conservatively. Our results regarding the incidence of new fractures are comparable to previous reports. Çevik et al. found that 23.3% of patients developed new fractures after kyphoplasty, strongly associated with poor bone quality and paraspinal muscle degeneration [33]. In contrast, Özgen et al. suggested that combining kyphoplasty with posterior dynamic stabilization may mitigate these risks, reducing fracture progression and improving functional outcomes [34]. These studies have supported the finding that patients treated with KP/VP require a shorter duration of brace use and experience a faster recovery process compared to those receiving conservative treatment.

The analysis of vertebral augmentation biomechanics requires thorough evaluation. The risk of adjacent vertebrae stress probabilities (OR = 3.10 according to our research) [20]. The analysis requires separate evaluation, and disrupted biomechanical stability increases when cement leaks into intervertebral disks, which results in higher fracture rates of adjacent and remote segment fractures because adjacent fractures occur when cement enters the disk space and disrupts load distribution, but remote fractures develop from low bone density and systemic osteoporosis [20]. The research indicates that better patient selection and improved surgical techniques, which manage cement amounts and prevent disk leakage, will produce superior long-term results. The implementation of regular anti-osteoporotic treatment after procedures leads to decreased secondary fracture occurrence, and healthcare providers should prioritize this treatment approach in post-procedural care [20].

The clinical outcomes and complication rates observed in our study were generally consistent with the results of similar studies in the literature. KP is seen to be a more effective option than conservative treatment in terms of pain management and functional status improvements. These results highlighted the importance of considering KP in the treatment planning process for patients.

This study had some limitations. The study was designed retrospectively, and no randomization or blinding techniques were employed, which may limit the generalizability and reliability of the results. As it was conducted at a single center with a one-year follow-up period, the study does not provide sufficient data on long-term outcomes. The study only used MRI to evaluate patients who showed symptoms, but did not perform routine scans, which might have resulted in missing hidden compression fractures. Furthermore, since patients were selected based on specific inclusion and exclusion criteria, the results may not be generalizable to the broader patient population. Future multicenter studies with extended follow-up durations may offer a more comprehensive perspective on the efficacy and safety of KP and VP procedures.

## 5. Conclusions

In this study, the clinical outcomes and complication rates of patients treated with kyphoplasty/vertebroplasty and those receiving conservative treatment were compared. The results revealed no significant difference in the incidence of new fractures between the surgical and conservative treatment groups (15.4% vs. 17.9%, *p* = 0.678). The surgical treatment group achieved superior pain management results through a mean VAS score difference of −0.8 (95% CI: −1.2 to −0.4, *p* = 0.032) and ODI improvement difference of 0.6 (95% CI: 0.3 to 0.9, *p* = 0.047) when compared to conservative treatment. The surgical group showed better results in terms of complication rates at 7.7% compared to 11.9 ± 0.5 months compared to 6.0 ± 1.0 months (*p* = 0.435), and patients recovered faster with their brace use reduced by 50%. The development of new fractures depended on three factors: patients who were older than others (adjusted OR: 2.48, 95% CI: 1.20–5.13), patients with low bone density (adjusted OR: 0.31, 95% CI: 0.15–0.63) and patients who experienced cement leakage during the procedure (adjusted OR: 3.10, 95% CI: 1.21–7.99). The study showed that patients who spent time outdoors and received anti-osteoporosis treatment experienced better outcomes. The research showed that kyphoplasty and vertebroplasty provided substantial pain relief and improved daily function while enabling patients to resume their activities more quickly. Future research involving multiple centers and extended observation periods with standard imaging checks will generate complete information about these treatment approaches.

## Figures and Tables

**Table 1:** Comparison of demographic and baseline characteristics between treatment groups.

Variables	Kyphoplasty/Vertebroplasty Group (*n* = 65)	Conservative Treatment Group (*n* = 67)	*p*-Value
Age (years, mean ± SD)	70.2 ± 8.5	71.3 ± 9.0	0.352
Sex (female, *n*, %)	37 (56.9%)	37 (55.2%)	0.837
Sex (male, *n*, %)	28 (43.1%)	30 (44.8%)	0.837
Body Mass Index (kg/m^2^)	25.4 ± 3.6	24.9 ± 3.8	0.547
Bone Mineral Density (T-score)	−2.8 ± 0.5	−2.7 ± 0.6	0.412
History of falls (yes, %)	15 (23.1%)	18 (26.9%)	0.589
Fracture location (T11-L2, %)	48 (73.8%)	48 (71.6%)	0.782
Number of fractured levels	1.3 ± 0.5	1.4 ± 0.6	0.298
Time to treatment (days)	5.2 ± 2.1	5.8 ± 2.4	0.124
Comorbidities (≥2, %)	42 (64.6%)	45 (67.2%)	0.758

**Table 2:** Clinical outcomes and complication rates with 95% confidence intervals.

Outcomes	Kyphoplasty/Vertebroplasty Group (*n* = 65)	Conservative Treatment Group (*n* = 67)	*p*-Value	Mean Difference [95% CI]
Hospital readmission, *n* (%) [95% CI]	5 (7.7%) [2.5–17.0]	7 (10.4%) [4.3–20.3]	0.548	−2.7% [−11.8 to 6.4]
Infection rate, *n* (%) [95% CI]	2 (3.1%) [0.4–10.7]	1 (1.5%) [0.0–8.0]	0.601	1.6% [−4.1 to 7.3]
Postoperative pain (VAS) [95% CI]	3.2 ± 1.0 [2.9–3.5]	4.0 ± 1.2 [3.7–4.3]	0.032	−0.8 [−1.2 to −0.4]
Mobility improvement (ODI) [95% CI]	4.5 ± 0.8 [4.3–4.7]	3.9 ± 0.9 [3.7–4.1]	0.047	0.6 [0.3 to 0.9]
Quality of life score [95% CI]	6.7 ± 1.1 [6.4–7.0]	5.9 ± 1.3 [5.6–6.2]	0.041	0.8 [0.4 to 1.2]
New fractures, *n* (%) [95% CI]	10 (15.4%) [7.6–26.5]	12 (17.9%) [9.6–29.2]	0.678	−2.5% [−13.7 to 8.7]
Median time to new fracture (months) [IQR]	4.5 [3–7]	5.0 [3–8]	0.543	-
Overall complications, *n* (%) [95% CI]	5 (7.7%) [2.5–17.0]	8 (11.9%) [5.3–22.2]	0.435	−4.2% [−13.4 to 5.0]

**Table 3:** Multivariable logistic regression of risk factors potentially associated with new fracture development.

Risk Factors	Adjusted Odds Ratio (OR)	95% Confidence Interval (CI)	*p*-Value
Age (per 10 years)	2.48	1.20–5.13	0.031
Bone Mineral Density (per unit decrease)	0.31	0.15–0.63	<0.001
Outdoor Activity (yes vs. no)	0.38	0.20–0.73	0.001
Cement Leakage (yes vs. no)	3.10	1.21–7.99	0.019
Anti-Osteoporosis Treatment (yes vs. no)	0.17	0.04–0.79	0.024

## Data Availability

The data presented in this study are available from the corresponding author upon reasonable request in accordance with ethical and privacy restrictions.

## Abbreviations

The following abbreviations are used in this manuscript:

OVCFs	Osteoporotic Vertebral Compression Fractures
VP	Vertebroplasty
KP	Kyphoplasty
MRI	Magnetic Resonance Imaging
CT	Computed Tomography
BMD	Bone Mineral Density
VAS	Visual Analog Scale
ODI	Oswestry Disability Index
